# Decline in the number of patients with meningitis in German hospitals during the COVID-19 pandemic

**DOI:** 10.1007/s00415-022-11034-w

**Published:** 2022-03-22

**Authors:** Stefanie Völk, Markus Pfirrmann, Uwe Koedel, Hans-Walter Pfister, Thomas Lang, Franziska Scheibe, Farid Salih, Julia Herzig-Nichtweiss, Julian Zimmermann, Angelika Alonso, Matthias Wittstock, Andreas Totzeck, Patrick Schramm, Ingo Schirotzek, Oezguer A. Onur, Johann Otto Pelz, Caroline Ottomeyer, Sebastian Luger, Kristian Barlinn, Tobias Binder, Gabriele Wöbker, Gernot Reimann, Christian Urbanek, Jan Heckelmann, Piergiorgio Lochner, Martin Berghoff, Silvia Schönenberger, Bernhard Neumann, Wolf-Dirk Niesen, Christian Dohmen, Hagen B. Huttner, Albrecht Günther, Matthias Klein

**Affiliations:** 1 Department of Neurology, University Hospital, LMU Munich, Marchioninistr. 15, 81377 Munich, Germany; 2 Institut für Medizinische Informationsverarbeitung, Biometrie und Epidemiologie, Ludwig-Maximilians-Universität, 81377 Munich, Germany; 3 Department of Neurology, NeuroCure Clinical Research Center, Charité-Universitätsmedizin Berlin, Charitéplatz 1, 10117 Berlin, Germany; 4 Department of Neurology, Charité-Universitätsmedizin Berlin, Campus Virchow-Klinikum, Augustenburger Platz 1, 13353 Berlin, Germany; 5 Department of Neurology, Charité-Universitätsmedizin Berlin, Charitéplatz 1, 10117 Berlin, Germany; 6 Department of Neurology, University Hospital Bonn, Venusberg-Campus 1, 53127 Bonn, Germany; 7 Department of Neurology, Medical Faculty Mannheim and Mannheim Center for Translational Neurosciences, University of Heidelberg, Mannheim, Germany; 8 Department of Neurology, University Medicine Rostock, Gehlsheimer Str. 20, 18147 Rostock, Germany; 9 Department of Neurology and Center for Translational Neuro- and Behavioral Sciences (C-TNBS), University Hospital Essen, University of Duisburg-Essen, Essen, Germany; 10 Department of Anaesthesiology, University Medical Center of the Johannes Gutenberg-University, Mainz, Germany; 11 Department of Neurology, University Hospital Giessen, Klinikstrasse 33, 35385 Giessen, Germany; 12 Klinik für Neurologie und Neurointensivmedizin, Klinikum Darmstadt, Grafenstrasse 9, 64283 Darmstadt, Germany; 13 Department of Neurology, Faculty of Medicine and University Hospital Cologne, University of Cologne, Cologne, Germany; 14 Department of Neurology, University Hospital Leipzig, Leipzig, Germany; 15 Hospital Agatharied, Agatharied, Germany; 16 Department of Neurology, Goethe-University Hospital Frankfurt, Schleusenweg 2-16, 60528 Frankfurt am Main, Germany; 17 Department of Neurology, University Hospital Carl Gustav Carus, Technische Universität Dresden, Dresden, Germany; 18 Department of Neurology, Julius-Maximilians-University, Würzburg, Germany; 19 Department of Intensive Care Medicine, Helios-Universitätsklinikum Wuppertal, Universität Witten-Herdecke, Wuppertal, Germany; 20 Department of Neurology, Klinikum Dortmund, Dortmund, Germany; 21 Department of Neurology, Hospital Ludwigshafen, Ludwigshafen, Germany; 22 Department of Neurology, University Hospital, Rheinisch-Westfälische Technische Hochschule Aachen, Aachen, Germany; 23 Department of Neurology, Saarland University Medical Center, Homburg, Germany; 24 Department of Neurology, Heidelberg University Hospital, Heidelberg, Germany; 25 Department of Neurology, University of Regensburg, Bezirksklinikum Regensburg, Regensburg, Germany; 26 Present Address: Department of Neurology, Donau-Isar-Klinikum Deggendorf, Deggendorf, Germany; 27 Department of Neurology and Neuroscience, Medical Center-University of Freiburg, 79106 Freiburg, Germany; 28 Department of Neurology, LVR-Klinik Bonn, Bonn, Germany; 29 Department of Neurology, Friedrich-Alexander-Universität, Erlangen-Nürnberg, Germany; 30 Department of Neurology, Jena University Hospital, Am Klinikum 1, 07747 Jena, Germany; 31 Emergency Department, University Hospital, LMU Munich, Marchioninistr. 15, 81377 Munich, Germany

**Keywords:** Meningitis, COVID-19 pandemic, Streptococcus pneumoniae, Enterovirus

## Abstract

**Background and objectives:**

In 2020, a wide range of hygiene measures was implemented to mitigate infections caused by the severe acute respiratory syndrome coronavirus 2 (SARS-CoV-2). In consequence, pulmonary infections due to other respiratory pathogens also decreased. Here, we evaluated the number of bacterial and viral meningitis and encephalitis cases during the coronavirus disease 2019 (COVID-19) pandemic.

**Methods:**

In a multicentre retrospective analysis of data from January 2016 until December 2020, numbers of patients diagnosed with bacterial meningitis and other types of CNS infections (such as viral meningitis and encephalitis) at 26 German hospitals were studied. Furthermore, the number of common meningitis-preceding ear-nose-throat infections (sinusitis, mastoiditis and otitis media) was evaluated.

**Results:**

Compared to the previous years, the total number of patients diagnosed with pneumococcal meningitis was reduced (*n* = 64 patients/year in 2020 vs. *n* = 87 to 120 patients/year between 2016 and 2019, all *p* < 0.05). Additionally, the total number of patients diagnosed with otolaryngological infections was significantly lower (*n* = 1181 patients/year in 2020 vs. *n* = 1525 to 1754 patients/year between 2016 and 2019, all *p* < 0.001). We also observed a decline in viral meningitis and especially enterovirus meningitis (*n* = 25 patients/year in 2020 vs. *n* = 97 to 181 patients/year between 2016 and 2019, all *p* < 0.001).

**Discussion:**

This multicentre retrospective analysis demonstrates a decline in the number of patients treated for viral and pneumococcal meningitis as well as otolaryngological infections in 2020 compared to previous years. Since the latter often precedes pneumococcal meningitis, this may point to the significance of the direct spread of pneumococci from an otolaryngological focus such as mastoiditis to the brain as one important pathophysiological route in the development of pneumococcal meningitis.

**Supplementary Information:**

The online version contains supplementary material available at 10.1007/s00415-022-11034-w.

## Introduction

The incidence of bacterial meningitis has decreased in Europe and North America during the last 30 years [13]. The main reason for this positive development is the success of vaccination strategies against *Haemophilus influenzae* during the early 1990s, *Neisseria meningitidis* shortly after the turn of the century, and *Streptococcus pneumoniae* approximately 10 years ago. Nevertheless, serotype replacement of strains that are not covered by the current vaccines against *Streptococcus pneumoniae* remains a concern, possibly leading to a resurgence of pneumococcal meningitis—the currently most common form of community-acquired bacterial meningitis in adults (followed by meningococcal meningitis and meningitis caused by *Listeria monocytogenes*) [6, 12]. In almost half of the adult cases, pneumococcal meningitis develops through local invasion of bacteria from an infection of the mastoid/middle ear or the paranasal sinuses [5, 30]. In 2020, a wide range of hygiene measures was implemented during the coronavirus disease 2019 (COVID-19) pandemic that especially aimed at the prevention of droplet infections and included the recommendation for community masks, social distancing and universal hygiene precautions. Moreover, public life was restricted, the home office was established, and schools were (partially) closed. These measures resulted not only in mitigation of infections caused by severe acute respiratory syndrome coronavirus 2 (SARS-CoV-2), but also in decreased numbers of seasonal influenza [24, 26]. In addition, the number of isolates of several other invasive infectious diseases that were reported to national reference libraries have declined [3, 17, 31]. Up to now, it is unclear if the implemented hygiene measures also reduced the number of meningitis cases. Here, we investigated the number of patients with bacterial meningitis who were treated at 26 German hospitals. Furthermore, the number of patients treated for other types of CNS infections (such as viral meningitis and encephalitis) and otolaryngological infections (sinusitis, mastoiditis and otitis media) were studied.

## Methods

This study is a multicentre retrospective analysis including data from 26 German hospitals from January 2016 until December 2020. Details on all study centres are listed under supplementary material. The enrolled study centres are located all across Germany (in 10 of 16 federal states) and the majority of the study centres included data from paediatric departments. The study population covers approximately 5–10% of the yearly hospital in-patients of Germany, more than 5% of all hospital beds and at least 10% of all intensive care beds in Germany (compared to data from the federal statistical office of Germany). Less than 2% of all German hospitals, but about 26% of the hospitals with more than 800 beds (which are usually tertiary care hospitals) and more than 50% (21 of 38) of the medical universities of Germany are included.

For each study centre, the yearly numbers of patients who were treated as in-patients with one of the following diagnoses as coded by one of the following International Classification of Disease (ICD) codes were determined: any bacterial meningitis and meningoencephalitis (G00, G01*, G04.2, G05.0), pneumococcal meningitis (G00.1), Listeria meningitis (A32.1), meningococcal meningitis (A39.0), viral meningitis/meningoencephalitis und myelitis (G02.0, A87.8, A87.9, G05.1*), enterovirus meningitis (A87.0), meningitis and encephalitis caused by herpes viruses (B00.4, B00.3), Varicella zoster virus meningitis (B02.1), mastoiditis (H70), sinusitis (J01), otitis media (H66) and COVID-19 (U07.1). As non-infectious controls, subarachnoid haemorrhage (I60) and intracranial haemorrhage (I61) were investigated. Subgroups were analysed for different age groups (< 18 years, 18–65 years, > 65 years). The study was approved by the ethics committee of the Ludwig-Maximilians-University (LMU) Munich (project number 21-0104 KB) and all responsible local ethics committees of the participating hospitals.

For statistics, an exact two-sided Poisson test (central method) [14] using the programming software R and the package “exactci” provided by Fay was used [8]. The ratio of two Poisson rates to be compared was calculated. The null hypothesis was that the Poisson rates are identical; the alternative hypothesis was that they are different. For the diseases under investigation, the number of events per year was known. The number of people at risk of experiencing an event in a certain year was unknown, but it was reasonable to assume that the risk could be attributed to the same number of people living in the area around the hospitals. Accordingly, the denominators of the Poisson rates cancelled each other out when we calculated the rate ratio. Technically, to run the package, a denominator must be entered. We always used one million, as there are no statistically relevant changes in the *p* value if, for instance, 10 million was used. For each entity, we compared data from 2020 to each of the four previous years. If at least one comparison showed no significant difference, no overall significance was recorded. Of course, all differences had to be in the same direction. In the case that both patient numbers were ≤ 5, no testing was performed. For each Poisson rate ratio, 95-percent confidence interval and *p* value were calculated. A *p* value of < 0.05 was considered significant.

## Results

### Pneumococcal meningitis in adults

**Fig. 1:**
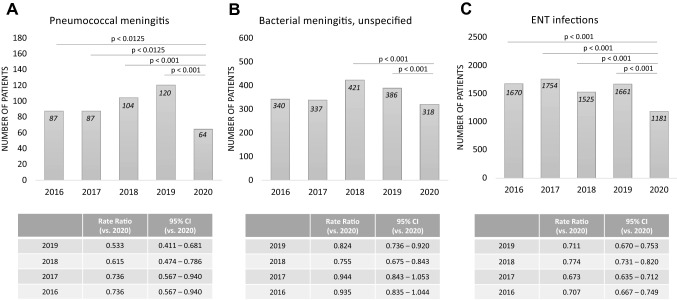
Bacterial meningitis and ENT infections. Numbers of patients diagnosed with **A** pneumococcal meningitis, **B** unspecified bacterial meningitis, and **C** ear-nose-throat (ENT, including mastoiditis, sinusitis or otitis media) infections from 2016 to 2020

For bacterial meningitis caused by *Streptococcus pneumoniae*, numbers for 2020 were significantly lower than in the four previous years (Fig. 1 A). In a subgroup analysis, the number of pneumococcal meningitis cases was significantly lower in 2020 than in the previous years for all patients > 18 years (*n* = 51 in 2020 vs. *n* = 79 in 2019, *n* = 73 in 2018, *n* = 61 in 2017, and *n* = 59 in 2016; 2019 *p* < 0.001, 2018 *p* < 0.001, 2017 *p* = 0.001, and 2016 *p* = 0.005). This was also true for patients older than 65 years (*n* = 13 in 2020 vs. *n* = 41 in 2019, *n* = 31 in 2018, *n* = 26 in 2017, and *n* = 28 in 2016; 2019 *p* < 0.001, 2018 *p* < 0.001; 2017 *p* = 0.008, and 2016 *p* = 0.003) (Table 1) but did not show significance in all four statistical tests in the age group 18 years to 65 years. There was no overall reduction of referred patients with pneumococcal meningitis from other hospitals to our study centres in 2020 compared to the previous years (data from 14 study centres available; *n* = 16 in 2020, *n* = 24 in 2019, *n* = 27 in 2018, *n* = 18 in 2017, *n* = 12 in 2016). For meningococcal meningitis, no significant reduction and rather low patient numbers were observed in all of the years (*n* = 9 cases in 2020 vs. *n* = 13 in 2019, *n* = 29 in 2018, *n* = 13 in 2017, and *n* = 29 in 2016). Similarly, for listeria meningitis, no significant reduction was seen (*n* = 13 patients in 2020, *n* = 15 in 2019, *n* = 22 in 2018, *n* = 18 in 2017, and *n* = 21 in 2016). Also for cases of bacterial meningitis that were not further specified, we could not observe a general reduction during the COVID pandemic in 2020 (Fig. 1 B).

**Table 1:**
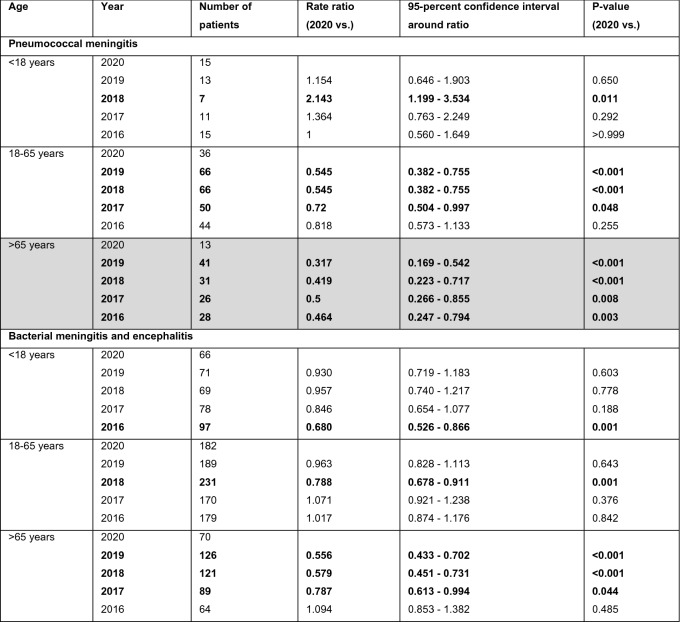
Bacterial meningitis

Numbers of patients diagnosed with bacterial meningitis/encephalitis and pneumococcal meningitis from 2016 to 2020. Bold print indicates a significant difference (*p* < 0.05) of 2020 compared to the other years. Grey background indicates that all tests of 2020 vs. 2019 to 2016 showed significant differences. For the Poisson rate of all years, the number of cases (events) was related to one million people at risk

### Sinusitis, otitis media and mastoiditis

Looking at major ear-nose-throat (ENT) infections that are known to precede pneumococcal meningitis in adults, a significant reduction was found in the number of patients who were treated for sinusitis, otitis media, or mastoiditis (Fig. 1 C). In subgroup analysis, the number of patients with sinusitis and otitis media was significantly lower in 2020 compared to the four previous years in patients from 18 to 65 years and in children and for mastoiditis in children (Table 2).

**Table 2:**
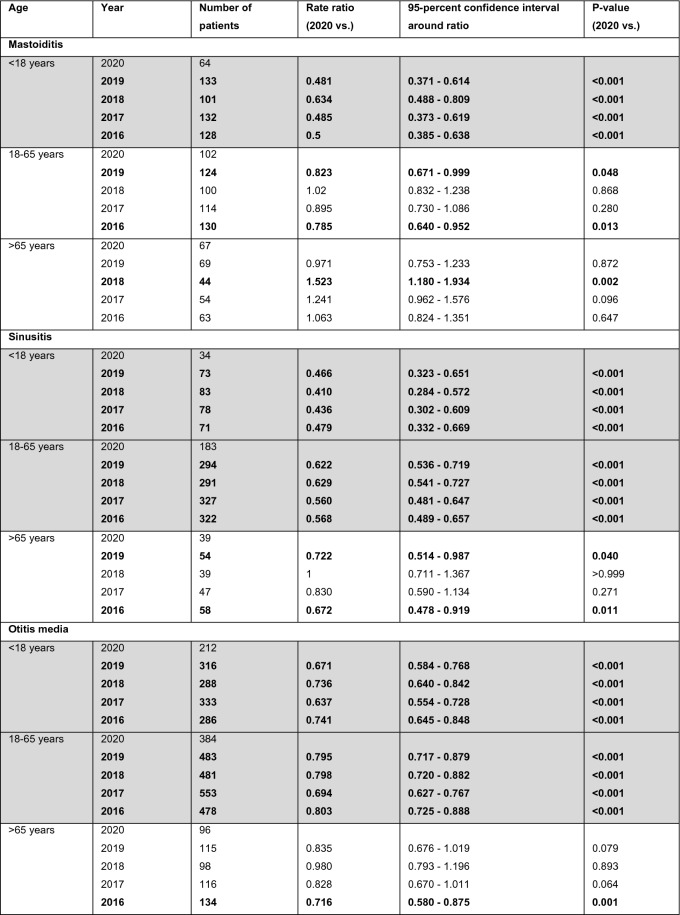
ENT Infections

Numbers of patients diagnosed with mastoiditis, sinusitis and otitis media from 2020 to 2016. Bold print indicates a significant difference (*p* < 0.05) compared to data of 2020. Grey background indicates that the entire hierarchical test approach from 2020 vs. 2019 to 2016 showed significant differences. For the Poisson rates of all years, the number of cases (events) was related to one million people at risk

### Viral meningitis

**Fig. 2:**
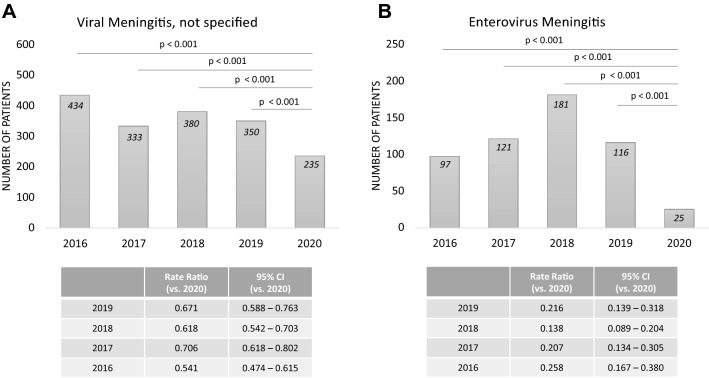
Viral meningitis. Numbers of patients diagnosed with **A** viral meningitis not further specified (excluding enterovirus, HSV and VZV) and **B** enteroviral meningitis

The number of patients diagnosed with viral meningitis was significantly lower in 2020 compared to the previous years (Fig. 2; 2020 *n* = 235, 2019 *n* = 350, 2018 *n* = 380, 2017 *n* = 333, 2016 *n* = 434), particularly in children and in patients between 18 and 65 years (Table 3). A strong decline was detected for patients diagnosed with enterovirus meningitis (Fig. 2 B; 2020 *n* = 25, 2019 *n* = 116, 2018 *n* = 181, 2017 *n* = 121, 2016 *n* = 97). In patients older than 65 years, patient numbers were low (Table 3). Furthermore, a significant reduction in the number of patients diagnosed with any other viral meningitis (excluding enterovirus, herpes virus and Varicella zoster virus (VZV) meningitis) was seen in 2020 compared to the four previous years (Fig. 2A). In patients with viral encephalitis/myelitis/encephalomyelitis, herpes virus or VZV meningitis, no significant reduction of numbers could be observed (Table 3).

**Table 3:**
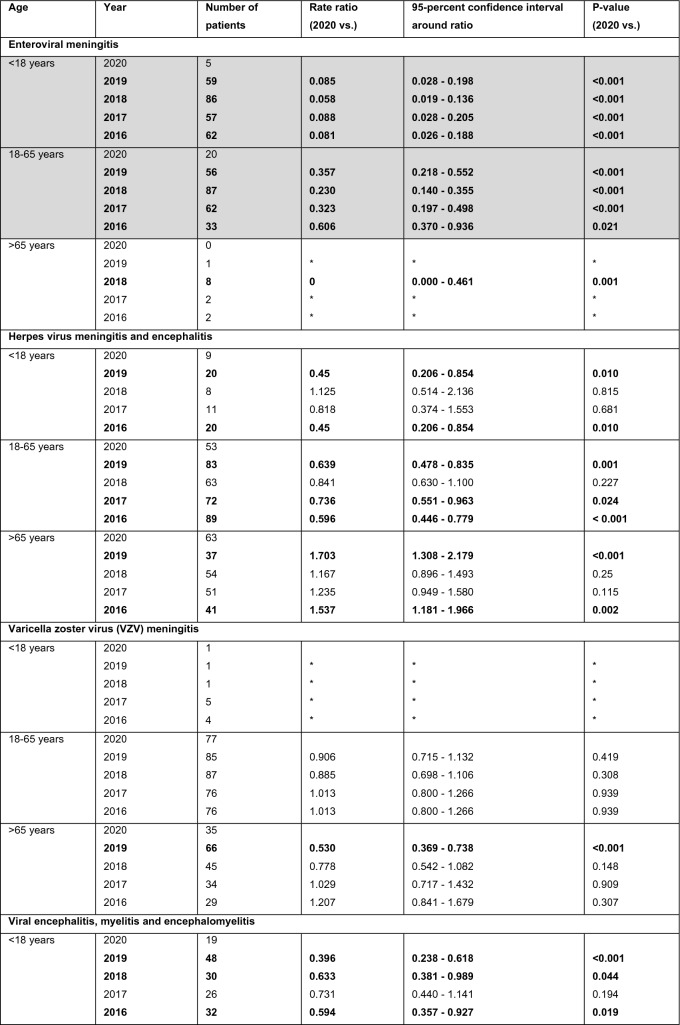

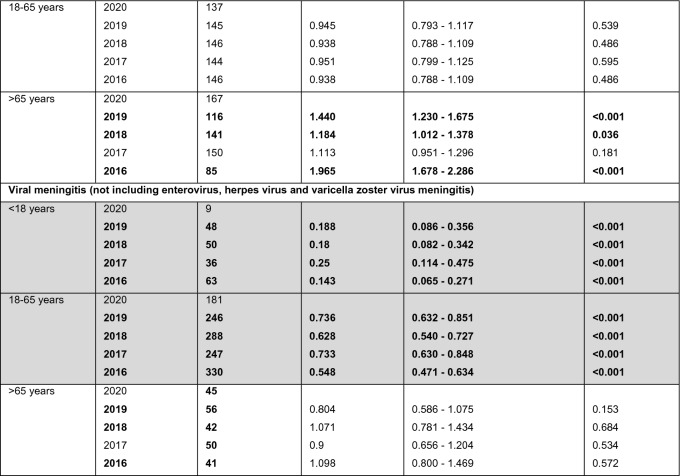
Viral meningitis and encephalitis

Numbers of patients diagnosed with enteroviral meningitis, herpes virus meningitis/encephalitis/encephalomyelitis, Varicella zoster virus (VZV) meningitis, viral myelitis/encephalitis/encephalomyelitis and viral meningitis. Bold print indicates a significant difference (*p* < 0.05) compared to data of 2020. Grey background indicates that the entire hierarchical test approach from 2020 vs. 2019 to 2016 showed significant differences. For the Poisson rates of all years, the number of cases (events) was related to one million people at risk. *No comparison between the numbers of cases was performed, because both numbers were less or equal 5

During the COVID-19 pandemic, a possible reduction of patient referrals to large hospitals due to limited ICU capacities might have affected the numbers of patients with pneumococcal meningitis at our study centres. However, numbers of patients with subarachnoid haemorrhage and intracranial haemorrhage (which are also potentially life-threatening neurological diseases that are usually treated at specialised neurological and neurosurgical intensive care centres in Germany) were not significantly altered (data from 15 study centres available): we did not find a significant reduction of cases with subarachnoid haemorrhage (*n* = 1260 in 2020, *n* = 1308 in 2019, *n* = 1314 in 2018, *n* = 1293 in 2017, *n* = 1207 in 2016, all *p* > 0.05) or intracranial haemorrhage (*n* = 3195 in 2020, *n* = 3141 in 2019, *n* = 3214 in 2018, *n* = 3150 in 2017, and *n* = 3084 in 2016). The number of patients treated for COVID-19 in 2020 was comparable between most study centres (see Supplemental Table 1).

## Discussion

The major findings of our study were: Compared to the previous years, in 2020 (1) the number of adult patients (but not children) diagnosed with pneumococcal meningitis, (2) the number of ENT infections, and (3) the number of viral meningitis and especially enterovirus meningitis were significantly reduced. A reduction of patient numbers was not observed for patients diagnosed with (4) bacterial meningitis not further specified, (5) viral myelitis and encephalomyelitis, and (6) herpes virus or VZV meningitis.

The finding that the number of pneumococcal meningitis cases is strikingly reduced suggests that the hygiene measures that were established during the pandemic not only protected from SARS-CoV-2 and direct pulmonary infections but may have also reduced infections not regarded as primary droplet infections. There are several possible explanations for this observation. First, an overall decline of bacterial respiratory infections after the implementation of containment strategies was observed, including those infections caused by *Streptococcus pneumoniae* [2]. A recent surveillance study based on data from microbiology laboratories from 26 countries worldwide showed a reduction of main respiratory isolates such as *Streptococcus pneumoniae* and *Haemophilus influenzae* but also *Neisseria meningitidis* in the first weeks of the pandemic, shortly after containment measures were set in place [4]. Second, almost half of the adult patients with pneumococcal meningitis suffer from an infection of the mastoid or middle ear or a paranasal sinus. Here, we could indeed detect a significant decline of patients admitted for such ENT infections, which may support the pathophysiological sequence of direct spread of pneumococci from an ENT focus to the brain [30]. The fact that we observed a reduction of certain ENT infections in specific age groups and no reduction of pneumococcal meningitis in the same age group does not necessarily argue against this theory, as numbers in some groups were quite low (e.g. in children with pneumococcal meningitis). Furthermore, in neonates, infants and children, meningitis by *Streptococcus pneumoniae* is less frequent and bacterial meningitis is rather caused by pathogens acquired during birth contact, aspiration, colonisation of the nasopharynx and following the invasion of the bloodstream [23]. Therefore, it is not surprising that lower numbers of ENT infections in children in 2020 did not correspond with a reduction of bacterial meningitis in this age group. One study conducted in Israel indicates decreased numbers of bacterial meningitis in paediatric patients during the COVID-19 pandemic in 2020 [9]. However, in this study only 29 patients with “bacteremia/sepsis/meningitis” within three years were included and the relevant subgroup was heterogeneous. Third, it cannot be ruled out that a reduction of post-viral invasive bacterial superinfections might have contributed to a decrease of pneumococcal infections and meningitis cases, as the overall number of viral infections other than SARS-CoV-2 has dropped in 2020 [19, 28]. Fourth, vaccination rates in our study cohort remain unknown and it is possible that the strong reduction of pneumococcal meningitis in the age group > 65 years might have been influenced by an increased vaccination rate for *Streptococcus pneumoniae,* which was significantly higher in patients > 60 years in 2020 compared to the previous years [20]. In Germany, pneumococcus vaccination is recommended for everyone above the age of 60 years, for chronically ill patients and infants. Within these groups, vaccination rates are usually between 10 and 40% for adults and up to 80% for children. For *Neisseria meningitidis* and *Haemophilus influenzae,* vaccination rates are very low in adults, but, as a consequence of changed vaccination recommendations, are currently above 90% in children. Public debate and calls for vaccination against pneumococcal infection during the early phase of the pandemic might have increased awareness in risk groups and led to an increase of pneumococcal vaccination rates in patients older than 60 years by 30% [20].

For meningococcal meningitis, our data showed no general decrease. This is in contrast to local public health data indicating a drastic decrease of invasive meningococcal infections in Germany in 2020 [21]. In detail, only 138 cases of invasive meningococcal infections were reported in Germany in 2020, which is strikingly lower than in 2019 when 257 cases were reported (295 cases in 2018) [21, 22]. In line with this observation, two large studies on meningococcal surveillance data have shown a decrease of meningococcal isolates [4, 17]. This is not surprising, as meningococci typically spread via droplets and protection can be established e.g. by wearing face masks [1]. The fact that we did not find a statistically significant reduction is most likely due to the very small number of cases that were treated in the hospitals that participated in this study.

Our data support previous signs of a reduction of viral and particularly enteroviral meningitis during the COVID-19 pandemic [11, 15, 16, 27]. The major reason for reduced viral meningitis numbers might be that social distancing, masks and other hygiene measures aimed at SARS-CoV-2 also mitigated transmission of other viruses, such as enteroviruses that are similarly excreted in the respiratory tract and transmitted from person to person by aerosols. Furthermore, enterovirus is known to be transmitted via a faecal-oral route, which has also been addressed by the hygiene measures set in place plus (partial) closure of schools and daycare facilities [18]. In Germany, schools were completely closed from about mid-March to the end of April, often followed by several months of reduced physical attendance and persisting mandatory hygiene precautions (distancing measures and community masks) for the rest of the year. At universities, remote learning prevailed the whole year. All in all, there were only minor regional and short-term differences in the adoption of hygienic measures in 2020, which we do not expect to have influenced our cumulative data of one year. In contrast to the reduction of enterovirus meningitis, we observed stable numbers for meningitis/encephalitis caused by Herpes simplex virus (HSV) and VZV in adults, as these viruses are not classically dependent on an aerogen or faecal-oral transmission mode, but result from reactivation of previous infections. Also, numbers for viral myelitis and encephalomyelitis, which are mainly caused by HSV, VZV, tick-born encephalitis virus, Cytomegalovirus (CMV) and Epstein-Barr virus (EBV), remained stable. These control groups indicate that it is not likely that meningitis patients (who usually suffer from severe symptoms) avoided hospital referral or even rejected admission because they were afraid of nosocomial SARS-CoV-2 infection as previously reported for general hospital admissions or minor stroke [25, 29].

Our study suggests that the implementation of relatively simple hygiene measures not only reduces the threat of a certain infection in a pandemic but may also lead to a decline of other infections that are not considered to be directly contagious, such as pneumococcal meningitis. However, the price for some of the containment strategies that were set in place is high and it is not clear what measures were mainly responsible for the reduction of patients treated for pneumococcal meningitis. Although it may sound reasonable that certain actions (such as wearing masks and avoiding crowds) might be of advantage for individual patients at risk of invasive pneumococcal disease, it would not be proportionate to maintain general hygienic measures after the pandemic [7, 10].

The strength of our study is that a multicentre approach was used and 26 predominantly tertiary hospitals contributed data. However, there are several limitations to our study. We saw no reduction of patients with pneumococcal meningitis from other hospitals to our mainly tertiary study centres in 2020, but overall numbers were small. We cannot completely rule out that fewer patients were transferred to tertiary care hospitals for specialized ICU treatment during the pandemic (as ICU beds were limited in most hospitals). The stable numbers we observed for subarachnoid and intracranial haemorrhage argue against such a reduction of referrals for patient care at specialized neurological ICUs. These controls are also potentially life-threatening neurological diseases that are usually treated at specialised neurological and neurosurgical ICUs in Germany, similar to patients with pneumococcal meningitis, where we saw a decline at our predominantly tertiary care hospitals.

Unfortunately, it was not possible to control for local COVID-19 activity as the 2020 COVID-waves fluctuated throughout the country and the regional numbers of meningitis patients were too small for statistical subgroup analysis. Our study shows that the numbers of meningitis patients treated at the centres varied. Possibly, the number of patients was also too low to control for other factors that might have influenced patient flows from regional hospitals to tertiary care centres over the years—especially as some university hospitals were found to only treat individual patients with meningitis each year. One further factor that might have contributed to the differences is that we relied on ICD codes and primary patient data was not checked. Thus, it is possible that yearly and regional differences in the way patients were coded might have influenced our data, for example in cases where more than one ICD code was applicable, such as viral meningitis and enteroviral meningitis. Also, we did not control for risk factors of bacterial meningitis beyond age, such as immunosuppression, cancer, alcoholism or smoking (5). These factors, however, might have been of interest, as diagnostics and therapy for autoimmune and malignant diseases as well as addictive behaviour might have been affected by the pandemic. The scenario that sicker patients died of COVID-19 and therefore did not develop meningitis (competing risks) is possible. Unfortunately, we could not retrieve data from all patients in each hospital, and for six study centres, data was only available for the neurological departments. As this remained constant for the whole study period, we do not expect that this has influenced our comparative results. Finally, we did not have access to mortality data and are therefore unable to comment on whether the decrease of patients with pneumococcal meningitis was also associated with a reduction of mortality. Big data from national databases on the numbers of CNS infections are not available yet but should be evaluated in the future to see if our hypotheses hold true.

## Conclusion

In our multicentre study, we clearly found that the number of patients with viral and pneumococcal meningitis was reduced during the pandemic compared to the previous years. Similarly, the number of patients with infections of the ear or the paranasal sinuses were lower. Although causality cannot be concluded from our data, this suggests that a reduction of ENT infections through the establishment of containment measures during the pandemic might have led to a decline of severe bacterial meningitis cases.

## Supplementary Information

Below is the link to the electronic supplementary material.

Supplementary file1 (DOCX 19 KB)

Supplementary file2 (DOCX 29 KB)

## Data Availability

Anonymized original data will be made available from the authors by request from any qualified investigator.
