# Supplementary material for: Decline in the number of patients with meningitis in German hospitals during the COVID-19 pandemic
Source: J Neurol. 2022 Mar 22;269(7):3389–99. doi: 10.1007/s00415-022-11034-w (PMC8938731; doi:10.1007/s00415-022-11034-w)
Supplement: Supplementary file 1 — Supplementary file1 (DOCX 19 KB) [file 415_2022_11034_MOESM1_ESM.docx]

**Supplemental Table 1** Study centres with numbers of hospital beds and treated in-patients.

| **Study centre** | **Standard beds** | **Patients 2020** | **Patients 2019** | **Patients 2018** | **Patients 2017** | **Patients 2016** | **Total number of COVID Patients 2020** | **COVID patients per hospital bed per year in 2020** |
| --- | --- | --- | --- | --- | --- | --- | --- | --- |
| Aachen | 1588 | 34220 | 35902 | 36656 | 36394 | 35208 | 476 | 0.300 |
| Berlin (Charité) | 2901 | 91090 | 105844 | 104141 | 102172 | 103217 | 1718 | 0.592 |
| Bonn (UKB) | 1224 | 51215 | 50645 | 47078 | 46816 | 46379 | 343 | 0.280 |
| Cologne | 1454 | 55489 | 60099 | 59379 | 59824 | 58981 | 352 | 0.242 |
| Darmstadt | 988 | 37605 | 40932 | 39672 | 39062 | 38787 | 464 | 0.470 |
| Dortmund | 1408 | 54998 | 60566 | 59739 | 61782 | 62363 | 682 | 0.484 |
| Dresden | 1410 | 55877 | 58672 | 57101 | 57344 | 58445 | 758 | 0.538 |
| Essen/Ruhr | 1268 | 49908 | 55447 | 52091 | 54143 | 53768 | 1108 | 0.874 |
| Frankfurt a. M. | 1134 | 40571 | 49608 | 48948 | 47287 | 46207 | 727 | 0.641 |
| Freiburg | 1616 | 59338 | 73356 | 71469 | 69899 | 68814 | 609 | 0,377 |
| Hausham | 350 | 17419 | 19561 | 20187 | 19900 | 19274 | 277 | 0.791 |
| Homburg | 1331 | 49037 | 54195 | 54703 | 55751 | 55122 | 413 | 0.310 |
| Leipzig | 1451 | 52492 | 56917 | 55266 | 54266 | 53220 | 496 | 0.342 |
| Ludwigshafen | 970 | 33949 | 40388 | 39409 | 38856 | 39782 | 570 | 0.588 |
| Mainz | 1434 | 64776 | 65586 | 65878 | 65406 | 58517 | 366 | 0.255 |
| Mannheim | 1352 | 44847 | 49001 | 50744 | 50666 | 51337 | 385 | 0.285 |
| Munich (LMU) | 1527 | 69764 | 78128 | 78673 | 78311 | 79356 | 787 | 0.515 |
| Rostock | 1043 | 35959 | 39212 | 39054 | 39135 | 38877 | 71 | 0.068 |
| Wuerzburg | 1377 | 56388 | 61582 | 61356 | 60907 | 59495 | N/A | N/A |
| Wuppertal | 1078 | 48143 | 55779 | 59472 | 58667 | 60113 | 597 | 0.554 |
| Bonn (LVR)* | 50 | 2507 | 2818 | 2684 | 2849 | 2778 | 13* | 0.260* |
| Erlangen* | 68 | N/A | N/A | N/A | N/A | N/A | 34* | 0.5* |
| Giessen* | 66 | 2467 | 2420 | 2259 | 3220 | 3299 | 0* | 0* |
| Heidelberg* | 84 | 7813 | 8220 | 8377 | 8430 | 9122 | N/A | N/A |
| Jena* | 64 | 3590 | 3917 | 3815 | 3556 | 3502 | 0* | 0* |
| Regensburg* | 58 | 3107 | 3704 | 3284 | 3189 | 3097 | 20* | 0.345* |

Study centres with numbers of hospital beds and numbers of treated in-patients for each year from 2020 to 2016. See author affiliations for details. * data available only from patients treated at the neurological departments (as data for the complete hospital were not available). N/A indicates missing data.
